# Genome-Wide Association Analysis of Soluble ICAM-1 Concentration Reveals Novel Associations at the *NFKBIK*, *PNPLA3*, *RELA*, and *SH2B3* Loci

**DOI:** 10.1371/journal.pgen.1001374

**Published:** 2011-04-21

**Authors:** Guillaume Paré, Paul M. Ridker, Lynda Rose, Maja Barbalic, Josée Dupuis, Abbas Dehghan, Joshua C. Bis, Emelia J. Benjamin, Dov Shiffman, Alexander N. Parker, Daniel I. Chasman

**Affiliations:** 1 Center for Cardiovascular Disease Prevention, Brigham and Women's Hospital, Harvard Medical School, Boston, Massachusetts, United States of America; 2 Donald W. Reynolds Center for Cardiovascular Research, Brigham and Women's Hospital, Harvard Medical School, Boston, Massachusetts, United States of America; 3 McMaster University, Hamilton, Canada; 4 Human Genetics Center and Institute of Molecular Medicine, University of Texas Health Science Center at Houston, Houston, Texas, United States of America; 5 National Heart, Lung, and Blood Institute's and Boston University's Framingham Heart Study, Framingham, Massachusetts, United States of America; 6 Department of Biostatistics, Boston University School of Public Health, Boston, Massachusetts, United States of America; 7 Department of Epidemiology, Erasmus Medical Center, Rotterdam, The Netherlands; 8 The Netherlands Consortium on Healthy Aging (NCHA), Leiden, The Netherlands; 9 Cardiovascular Health Research Unit, Department of Medicine, University of Washington, Seattle, Washington, United States of America; 10 Section of Preventive Medicine and Epidemiology, Department of Medicine, Boston University School of Medicine, Boston, Massachusetts, United States of America; 11 Department of Epidemiology, Boston University School of Public Health, Boston, Massachusetts, United States of America; 12 Celera, Alameda, California, United States of America; 13 Amgen, Cambridge, Massachusetts, United States of America; University of Liège, Belgium

## Abstract

Soluble ICAM-1 (sICAM-1) is an endothelium-derived inflammatory marker that has been associated with diverse conditions such as myocardial infarction, diabetes, stroke, and malaria. Despite evidence for a heritable component to sICAM-1 levels, few genetic loci have been identified so far. To comprehensively address this issue, we performed a genome-wide association analysis of sICAM-1 concentration in 22,435 apparently healthy women from the Women's Genome Health Study. While our results confirm the previously reported associations at the *ABO* and *ICAM1* loci, four novel associations were identified in the vicinity of *NFKBIK* (rs3136642, P = 5.4×10^−9^), *PNPLA3* (rs738409, P = 5.8×10^−9^), *RELA* (rs1049728, P = 2.7×10^−16^), and *SH2B3* (rs3184504, P = 2.9×10^−17^). Two loci, *NFKBIB* and *RELA*, are involved in NFKB signaling pathway; *PNPLA3* is known for its association with fatty liver disease; and *SH3B2* has been associated with a multitude of traits and disease including myocardial infarction. These associations provide insights into the genetic regulation of sICAM-1 levels and implicate these loci in the regulation of endothelial function.

## Author Summary

Soluble Intercellular Adhesion Molecule 1 (sICAM-1) is an inflammatory marker that has been associated with several common diseases such as diabetes, heart disease, stroke, and malaria. While it is known that blood concentrations of sICAM-1 are at least partially genetically determined, our current knowledge of which genes mediate this effect is limited. Taking advantage of technologies allowing us to interrogate genetic variation on a whole-genome basis, we found that variation in the *NFKBIK*, *PNPLA3*, *RELA*, and *SH2B3* genes are important determinant of sICAM-1 blood concentrations. The *NFKBIB* and *RELA* genes are involved in regulation of inflammation. These observations are significant because this is the first report of genetic association within these extensively studied inflammation genes. The *PNPLA3* gene has previously been associated with liver disease, and the *SH2B3* gene has been associated with a multitude of traits including cardiovascular disease. Extension of these associations to sICAM-1 adds to the intriguing diversity of effects of these genes.

## Introduction

A member of the immunoglobulin superfamily of adhesion receptors, ICAM-1 is expressed on endothelial cells where it serves as a receptor for the leukocyte integrins LFA-1 and Mac-1 [1]. A soluble form of ICAM-1 (sICAM-1) is present in plasma and is thought to arise from proteolytic cleavage of the extra-cellular domains of ICAM-1. Although the physiologic function of soluble ICAM-1 remains to be fully defined, plasma concentration of sICAM-1 have a predictive value for the risk of myocardial infarction, ischemic stroke, peripheral arterial disease and noninsulin-dependent diabetes mellitus in epidemiological studies [2]–[4].

We recently described a genome-wide association study of sICAM-1 in 6,578 apparently healthy women from the Women's Genome Health Study (WGHS), which confirmed a known association at the ICAM1 locus and identified a novel association at the ABO locus [5]. These results were subsequently replicated in large-scale genomics studies from Barbalic [6] et al. and Qi [7] et al. Nevertheless, the total variance explained by these associations remained low (8.4%) as compared to the relatively high heritability estimates (from 0.34 to 0.59) [8], [9] for sICAM-1. We therefore hypothesized that other, weaker, common genetic determinants of sICAM-1 remained to be discovered. To explore this issue, we performed a larger genome-wide association study (GWAS), evaluating 334,295 SNPs in 22,435 apparently healthy women of European ancestry from the WGHS.

## Results

**Figure 1:**
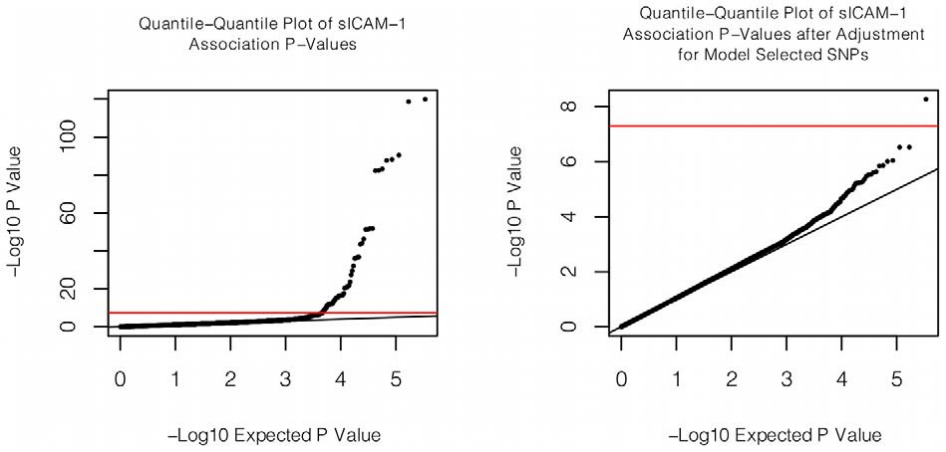
Quantile-quantile plot of association with sICAM-1. The quantile-quantile plot of sICAM-1 association P-values is shown on the left. On the right, the same quantile-quantile plot is shown, but after adjusting sICAM-1 values for the 9 SNPs retained by the model selection algorithm.

**Figure 2:**
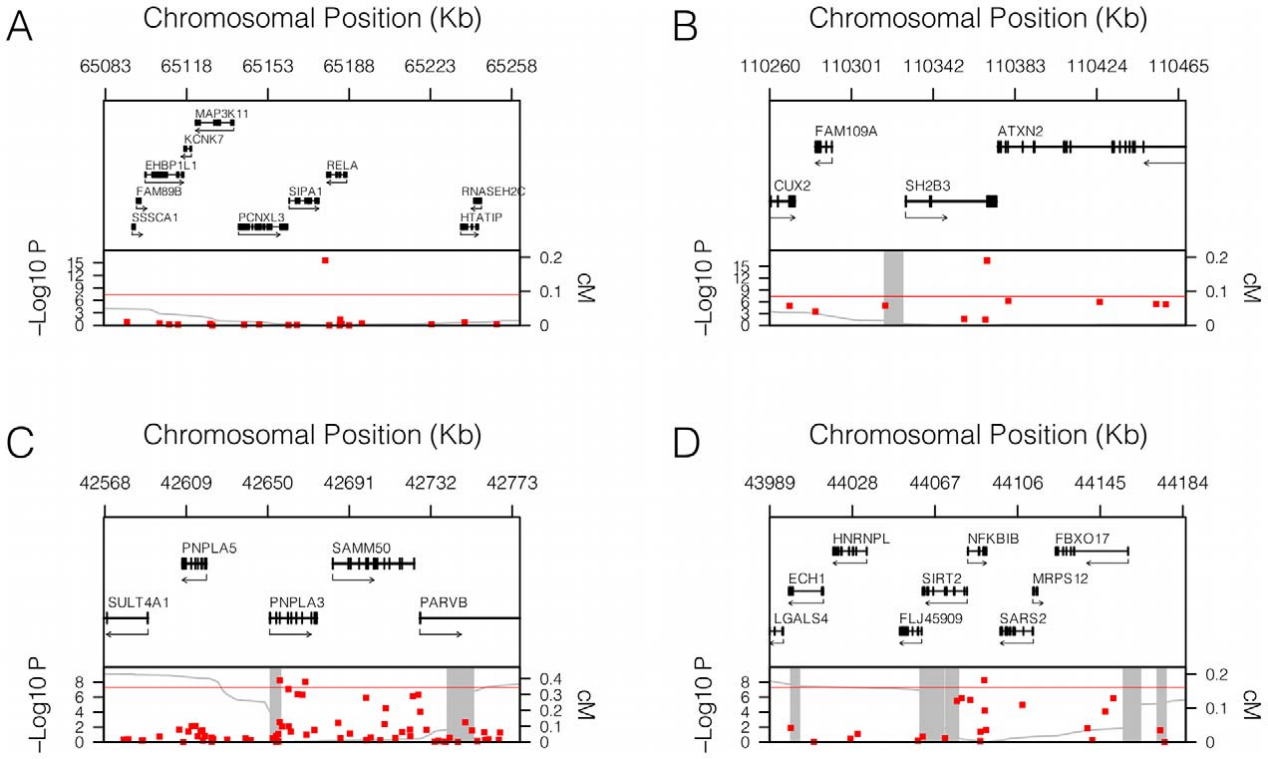
Genomic context of novel associations. Genomic context for each of the four novel loci with significant association with sICAM-1 concentration. (A) *RELA* locus (11q13.1); (B) *SH2B3* locus (12q24.12); (C) *PNPLA3* locus (22q13.31); and (D) *NFKBIB* locus (19q13.2). Upper panel: Genes from RefSeq release 25. Only one isoform is shown when multiple splicing variants are known. Lower Panel: SNPs are shown according to their physical location and –log_10_ P-values for association with sICAM-1 (red dots). The red line represents the genome-wide significance threshold of 5×10^−8^. Also shown is the genetic distance in cM from the lowest P-value SNP (light grey line) along with the position of recombination hotspots (light grey vertical bars). Recombination rates and hotspots are based on HapMap data, as described by McVean et al. [43] and Winckler et al. [44].

We found that 67 SNPs passed our pre-specified threshold of genome-wide significance of P<5×10^−8^ for association with sICAM-1 (Table S1 and Figure 1A). These SNPs clustered within 5 loci in the vicinity of *ABO* (9q34.2), *RELA* (11q13.1), *SH2B3* (12q24.12), *ICAM1* (19p13.2) and *PNPLA3* (22q13.31). The *ICAM1* [10], [11] and *ABO* [5] loci have previously been identified as contributing to sICAM-1 levels, but the *SH2B3*, *RELA* and *PNPLA3* loci were not previously shown to be associated with sICAM-1. The genomic context of these three latter loci is illustrated in Figure 2A, 2B and 2C.

In order to determine whether more than one non-redundant association signal could be detected at each of these five loci, we applied a model selection algorithm. The SNP with the lowest P-value for association was the only one retained at every locus with the exception of the *ICAM1* locus, where 5 SNPs were selected by the model (Table 1). Interestingly, model selected SNPs at the *ICAM1* locus showed lower P-value when they were all included in a single multivariate model than when considered separately. Three of the model selected SNPs at the *ICAM1* locus (rs281437, rs1801714 and rs11575074) were not significant at a genome-wide level of significance in a univariate analysis. We performed two analyses to determine if the multiple SNPs selected at the ICAM1 locus were the result of an underlying association with a known but untyped variant. First, we tested all imputed SNPs (using MACH) within 1.5 Mb of rs1799969 (the lead SNP at that locus) for association with adjusted sICAM-1 levels. No imputed SNP was more significant than the directly genotyped rs1799969. Second, we tested the same set of imputed SNPs after additional adjustment of sICAM-1 levels for the effect of model selected SNPs. No additional SNP was associated at genome-wide significance. The 5 SNPs at the ICAM1 locus selected by our algorithm were also used in haplotype analysis using WHAP [12], as implemented in PLINK [13] (Table 2). The estimate of the proportion of variance attributable to haplotypes, as well as their regression coefficients, is consistent with the linear model of these same SNPs, reinforcing the adequacy of an additive model to explain the association.

**Table 1:** SNPs retained by the model selection algorithm.

								Univariate Analysis		Multivariate Analysis	
SNP	Chr.	Position (Kb)	MAF	H-W P-Value	Minor Allele	Function	Nearest Gene	Beta (ng/mL)	P-Value	Beta (ng/mL)	P-Value
rs507666	9q34.2	135139.2	0.20	0.00072	A	intron	ABO	-17.3	3.0E-91	-16.8	4.2E-32
rs1049728	11q13.1	65177.7	0.06	0.79	C	3′ Untranslated	RELA	-11.5	2.7E-16	-11.2	3.2E-88
rs3184504	12q24.12	110369.0	0.49	0.01	T	coding-nonsynonymous	SH2B3	5.8	2.9E-17	5.4	4.2E-16
rs1799969	19p13.2	10255.8	0.11	0.50	A	coding-nonsynonymous	ICAM1	-24.9	1.3E-120	-41.5	1.3E-15
rs5498	19p13.2	10256.7	0.43	0.13	G	coding-nonsynonymous	ICAM1	13.8	5.7E-89	30.5	5.0E-249
rs1801714	19p13.2	10256.2	0.03	0.66	A	coding-nonsynonymous	ICAM1	8.0	5.9E-05	-12.2	2.5E-09
rs281437	19p13.2	10258.2	0.29	0.48	A	intron	ICAM1	-1.8	1.6E-02	7.6	7.2E-16
rs11575074	19p13.2	10262.1	0.05	0.09	A	intron	ICAM5	7.3	1.8E-06	11.0	1.2E-11
rs3136642	19q13.2	44090.3	0.38	0.49	G	Intron	NFKBIB	-3.8	7.9E-08	-4.1	5.4E-09
rs738409	22q13.31	42656.1	0.22	0.00001	G	coding-nonsynonymous	PNPLA3	4.9	5.8E-09	5.0	6.4E-10

**Table 2:** Haplotype Analysis of rs1799969, rs1801714, rs5498, rs281437, and rs11575074 (19p13.2; ICAM1 locus).

Haplotype					Frequency	Beta (ng/mL)
rs1799969	rs1801714	rs5498	rs281437	rs11575074		
G	G	A	A	A	0.05	Reference
G	G	A	A	G	0.24	-10.46
G	A	G	G	G	0.03	-0.03
A	G	G	G	G	0.11	-29.55
G	G	G	G	G	0.29	12.21
G	G	A	G	G	0.27	-18.29

Omnibus (5df) P-value <10^−300^.

Next we tested whether any additional SNPs are associated with sICAM-1 levels after adjustment for the model selected SNPs (see Figure 1B). A single SNP was associated with sICAM-1 at genome-wide significance (P = 5.4×10^−9^; −4.1 ng/mL per minor allele) in the vicinity of the *NFKBIB* locus at 19q13.2 (Figure 2D). This SNP, rs3136642, is intronic to *NFKBIB* and had a minor allele frequency of 0.38. The model selection algorithm retained no other SNP at the *NFKBIB* locus. Further adjustment of sICAM-1 values for rs3136642 did not identify any additional SNP with genome-wide significant association with sICAM-1. We also performed GWAS analysis using imputed genotypes (using MACH). Because no new locus reached genome-wide significance after adjustment for model selected SNPs, only results of directly genotyped SNPs are presented. These results were essentially unchanged when the first 10 components of a principal component analysis were included as covariates to account for sub-Caucasian stratification. All 4 novel loci identified in WGHS were replicated (one-sided P<0.05) in 9,813 individuals from the CHARGE consortium [14] (Table 3).

**Table 3:** Replication of novel loci in CHARGE (N = 9,813).

SNP	Nearest Gene	Minor Allele in WGHS	Allele Frequency	Effect (log-sICAM-1)	Standard Error	P-value (one sided)
rs4802998*	NFKBIB	G	0.38	0.007	0.004	0.048
rs738409	PNPLA3	G	0.23	0.019	0.005	4.9 E-5
rs1049728	RELA	C	0.05	−0.063	0.016	3.7 E-5
rs3184504	SH2B3	T	0.50	0.015	0.004	1.2 E-4

*The *NFKBIB* SNP rs3136642 reported in WGHS was not available in CHARGE. Consequently, rs4802998 was chosen for replication as this SNP had the second strongest association P-value at this locus in WGHS (p = 1.3×10^−6^).

Collectively, the 5 SNPs at the *ICAM1* gene locus explained 6.5% of sICAM1 total variance, whereas the other loci explained from 0.1 to 1.4% of the variance. In comparison, clinical covariates explained 19.5% of the variance (Table 4). For 4 of the loci, there was no strong evidence for non-additive effects of the minor allele as judged by lack of significance for a likelihood ratio test comparing the additive regression model to an alternative genotype model with an additional degree of freedom. However, the non-additive component was significant for rs507666 (P = 9.3×10^−6^) at the ABO locus with a tendency toward a dominant effect (mean sICAM-1 of 362.1, 342.4 and 335.4 ng/mL for 0, 1 and 2 minor alleles, respectively). The *PNPLA3* SNP rs738409 also showed evidence of non-additive association (P = 4.6×10^−5^) with a tendency toward a recessive model (mean sICAM-1 of 352.8, 356.0 and 367.7 ng/mL for 0, 1 and 2 minor alleles, respectively). In spite of these non-additive trends, no additional locus reached genome-wide significance when a genotypic test, which does not assume an additive model of association, was conducted.

**Table 4:** Variance explained.

		Variance explained	Sub-Total
Clinical covariates	Age	0.0149	
	Menopause	0.0049	
	Smoking	0.1315	
	BMI	0.0436	0.1950
			
ABO	rs507666	0.0144	0.0144
			
RELA	rs1049728	0.0025	0.0025
			
SH2B3	rs3184504	0.0026	0.0026
			
ICAM1	rs1799969	0.0194	
	rs1801714	0.0002	
	rs5498	0.0394	
	rs281437	0.0040	
	rs11575074	0.0016	0.0646
			
NFKBIB	rs3136642	0.0012	0.0012
			
PNPLA3	rs738409	0.0014	0.0014
**TOTAL**			**0.2817**

Model selected SNPs were tested for association with other available inflammation markers (C-reactive protein and fibrinogen). No significant association was noted (P>0.01) after adjusting for multiple hypothesis testing. Model selected SNPs were also tested for association with incident cardiovascular events (myocardial infarction, coronary revascularization, stroke and total cardiovascular event) over a mean follow-up period of 14 years. A Cox proportional hazard model was used adjusting for age at study entry. Only the *SH2B3* SNP rs3184504 was associated with incident myocardial infarction (315 events), with each minor allele increasing the risk (P = 0.011; OR 1.23 95% CI 1.05–1.43). The association remained significant after further adjustment for sICAM-1 levels (P = 0.028; OR 1.20 95% CI 1.02–1.41). Given the known association of sICAM-1 with cardiovascular risk and the association of selected SNPs with sICAM-1, we estimated the power to detect an association between the *SH2B3* SNP rs3184504 and myocardial infarction to be 6%, for alpha = 0.05. In comparison, power varied from 5% (rs281437) to 11% (rs5498) for other SNPs. The *PNPLA3* SNP rs738409 was tested for association with triglyceride, LDL cholesterol, HDL cholesterol and BMI as this gene is known to be involved in lipid metabolism and association with BMI has been previously suggested [15]. No significant association was observed.

Since smoking accounts for a large fraction of the variation in sICAM-1 levels, we tested associated SNPs for interaction with smoking. A significant interaction was observed for the ICAM1 SNP rs1799969 (interaction P = 1.6×10^−9^) whereby current smokers had a stronger genetic association, as we previously reported [16]. A novel interaction was also observed with the ABO SNP rs507666, again with a stronger genetic association in current smokers (P = 0.0003). When restricting the GWAS analysis to current smokers, an additional association was observed with rs8034191 (P = 3.5×10^−8^). This latter SNP is located on chromosome 15 near the nicotinic acetylcholine receptor subunit genes CHRNA3 and CHRNA5. This locus is known to be associated with smoking behavior [17], [18] and rs8034191 has recently been associated with smoking quantity [19]. No novel association was observed when restricting the GWAS analysis to non-smokers after adjustment for the previously described loci.

**Figure 3:**
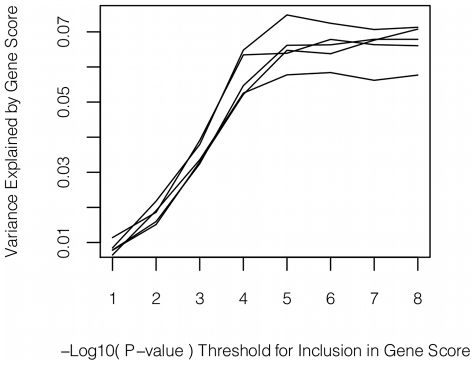
Polygene analysis. Variance explained (adjusted R^2^) by gene scores using varying P-value thresholds for inclusion of SNPs. Each P-value threshold was tested 5 times using a 5-fold cross-validation procedure.

We also tested whether multiple variants of individually weak effect could contribute to sICAM-1 levels. In cross-validation procedures, no increase in variance explained was observed when using P-value cut-offs less significant than 10^−8^ for inclusion of SNPs in gene scores (see Figure 3). In other words, selection of SNPs on the basis of P-value alone was not able to identify more of the genetic variance than could be explained by the SNPs with association P-value <10^−8^.

## Discussion

Six loci – *ABO*, *ICAM1*, *NFKBIK*, *PNPLA3*, *RELA* and *SH2B3* – have been identified in this report for association with sICAM-1. While the *ABO* [5] and *ICAM1* [10], [11] loci had been previously reported, we extended the number of non-redundantly associated variants at the ICAM1 locus by demonstrating association of rs11575074 and rs1801714 in multivariate analysis along with the known rs1799969, rs5498 and rs281437 SNPs [5]. Neither rs1801714 nor rs11575074 are predicted eQTL (http://eqtl.uchicago.edu/Home.html), but rs1801714 is a missense variant (P352L) and rs11575074 is located in a predicted binding site for several transcription factors including *PPARG* [20]. The *NFKBIK*, *PNPLA3*, *RELA* and *SH2B3* associations are novel. No strong contribution of weakly associated variants was observed in the polygene analysis whereby SNPs of varying statistical significance were included in gene scores.

Nuclear factor kB (NF-kB) proteins are a family of transcription factors involved in a number of physiological processes that include cell survival, proliferation, and activation. The NF-kB proteins (NFKB1 or NFKB2) are bound to REL, RELA, or RELB to form the NF-kB complex. These complexes are typically localized in the cytoplasm, where they are trapped by binding to IkB inhibitory proteins NFKBIA or NFKBIB. Upon inflammatory simulation, IkB kinase A and B phosphorylate IkB inhibitory proteins and mark them for degradation via the ubiquitination pathway, thereby allowing activation of the NF-kappa-B complex. Activated NF-kB complexes translocate into the nucleus and bind to NF-kB DNA binding motifs. NF-kB triggers transcription of various genes critical to inflammation, such as cytokines, chemokines and cell adhesion molecules including ICAM1 [21], [22]. Remarkably, two of the novel associations involve genes physically interacting with NF-kB. No genetic interaction, however, was noted between these two SNPs (data not shown). Taken together, these results emphasize the importance of the NFKB pathway in the regulation of sICAM-1 levels.

*PNPLA3* encodes a protein of unknown function that belongs to the patatin-like phospholipase family. Members of that family are believed to complement hormone sensitive lipase for adipocyte triacylglycerol lipase activity. The methionine allele of the missense *PNPLA3* SNP rs738409 (Ile148Met) has recently been associated with increased hepatic fat levels, hepatic inflammation and plasma levels of liver enzymes (traits linked to insulin resistance and obesity) [23], [24]. Nevertheless, rs738409 has been shown not to be associated with insulin resistance [25] although a previous study demonstrated an association with insulin secretion in response to oral glucose tolerance test [15]. Levels of the inflammatory marker sICAM-1 are known to be correlated with insulin resistance and obesity [4]. Consistent with rs738409 modulating the response to insulin resistance and associated phenotypes, the risk allele for fatty liver disease was associated with increased sICAM-1 levels.

*SH2B3* encodes Lnk, an adaptor protein that mediates the interaction between extra-cellular receptors, such as the T-cell receptor and the thrombopoietin receptor MPL, and intracellular signaling pathways. Cells from Lnk-deficient mice show an increased sensitivity to several cytokines and altered activation of the RAS/MAPK pathway in response to IL3 and stem cell factor [26]. The same *SH2B3* SNP rs3184504 identified in our study has previously been associated with multiple other traits, including blood pressure [27], [28], blood eosinophil number [29], myocardial infarction [29], celiac disease [30], type I diabetes [31], LDL-cholesterol [32], asthma [29], blood platelet number [33], hemoglobin concentration [34] and hematocrit [34]. Furthermore, rs3184504 is a non-synonymous SNP (Arg262Trp) whose derived allele (Trp) is part of a haplotype that has been suggested to have been introduced 3,400 years ago and selectively swept in European populations [33]. The derived allele is the risk allele for coronary artery disease and was the allele associated with higher sICAM-1 concentration. Association of rs3184504 with sICAM-1 further demonstrates the remarkable pleiotropy of that genetic variant by extending its effect to endothelial cell adhesion molecules. An interesting hypothesis is whether changes in sICAM-1 are mediated through increased sub-clinical atherosclerosis, but further studies will be needed to address this question.

In this report, we demonstrate genetic association of sICAM-1 with the *ABO*, *ICAM1*, *NFKBIK*, *PNPLA3*, *RELA* and *SH2B3* loci. These findings broaden our current knowledge of the genetic architecture of sICAM-1 with identification of four novel loci. The novel association at *PNPLA3* reinforces the importance of insulin resistance-related processes in the regulation of sICAM-1 levels. The observed associations also provide evidence of functional genetic variation at two genes – *NFKBIK* and *RELA* – well known for their implication in the NF-kB pathway, therefore providing a basis for the study of these polymorphisms in other conditions where this same pathway is involved. The results also extend the effect of the *SH2B3* SNP rs3184504 to endothelial function.

## Methods

### Ethics Statement

All analyses were performed with approval of the institutional review board of the Brigham and Women's Hospital. All members of the WGHS cohort were participants in the WHS who provided an adequate baseline blood sample for plasma and DNA analysis and who gave consent for blood-based analyses and long-term follow-up.

### Study Sample and sICAM-1 Measurements

All participants in this study were part of the Women's Genome Health Study (WGHS) [35]. Briefly, participants in the WGHS include North American women from the Women's Health Study (WHS) with no prior history of cardiovascular disease, diabetes, cancer, or other major chronic illness who also provided a baseline blood sample at the time of study enrollment. For all WGHS participants, EDTA anticoagulated plasma samples were collected at baseline and stored in vapor phase liquid nitrogen (−170°C). Circulating plasma sICAM-1 concentrations were determined using a commercial ELISA assay (R&D Systems, Minneapolis, Minn.); the assay used is known not to recognize the K56M (rs5491) variant of ICAM-1 [36] and the 82 Caucasian carriers of this mutation were therefore excluded from further analysis. The intra-assay coefficient of variation was 6.7% and the reported intra-individual coefficient of variation 7.6% [37]. This study has been approved by the institutional review board of the Brigham and Women's Hospital. Additional clinical characteristics of this sample are provided in Table S2.

### Genotyping

Samples were genotyped with the Infinium II technology from Illumina. Either the HumanHap300 Duo-Plus chip or the combination of the HumanHap300 Duo and I-Select chips was used. In either case, the custom content was identical and consisted of candidate SNPs chosen without regard to allele frequency to increase coverage of genetic variation with impact on biological function including metabolism, inflammation or cardiovascular diseases. Genotyping at 318,237 HumanHap300 Duo SNPs and 45,571 custom content SNPs was attempted, for a total of 363,808 SNPs. Genetic context for all annotations are derived from human genome build 36.1 and dbSNP build 126.

SNPs with call rates <90% were excluded from further analysis. Likewise, all samples with percentage of missing genotypes higher than 2% were removed. Among retained samples, SNPs were further evaluated for deviation from Hardy-Weinberg equilibrium using an exact method [38] and were excluded when the P-value was lower than 10^−6^. Samples were further validated by comparison of genotypes at 44 SNPs that had been previously ascertained using alternative technologies. SNPs with minor allele frequency >1% in Caucasians were used for analysis. After quality control, 334,295 SNPs were left for analysis.

### Population Stratification

Because population stratification can result in inflated type I error in a GWAS, a principal component analysis using 1443 ancestry informative SNPs was performed using PLINK [13] to confirm self-reported ancestry. Briefly, these SNPs were chosen based on Fst >0.4 in HapMap populations (YRB, CEU, CHB+JPT) and inter-SNP distance at least 500 kb in order to minimize linkage disequilibrium. Different ethnic groups were clearly distinguished with the two first components. 31 self-identified Caucasian women were removed from analysis because they did not cluster with other Caucasians, leaving 22,435 non-diabetic participants with non-missing sICAM-1 information for analysis. To rule out the possibility that residual stratification within Caucasians was responsible for the associations observed, a principal component analysis [39] was performed in Caucasians (only) using 64,205 SNPs chosen to have pair-wise linkage disequilibrium lower than r^2^ = 0.2. The first ten components were then used as covariates in the association analysis. As adjustment by these covariates did not change the conclusions, we present analysis among Caucasian participants without further correction for sub-Caucasian ancestry unless stated otherwise.

### Association Analysis

Plasma concentrations of sICAM-1 were adjusted for age, smoking, menopause and body mass index using a linear regression model in R to reduce the impact of clinical covariates on sICAM-1 variance. The adjusted sICAM-1 values were then tested for association with SNP genotypes by linear regression in PLINK [13], assuming an additive contribution of each minor allele. A conservative P-value cut-off of 5×10^−8^ was used to correct for the roughly 1,000,000 independent statistical tests thought to correspond to all the common genetic variation of the human genome [40], [41].

### Model Selection Algorithm

To investigate whether more than one SNP in each locus is independently associated with sICAM-1, a forward selection multiple linear regression model was used. For each locus with at least one genome-wide significant SNP (i.e. P<5×10^−8^), all genotyped SNPs within 1.5 Mb of the most significantly associated SNP and passing quality control requirements were selected for potential inclusion in our model. The forward selection algorithm then proceeded in two steps. In the first step, all SNPs not yet included in the multiple regression model were tested for association with sICAM-1. In step two, the SNP with the smallest P-value was included in the model if its multiple regression P-value was less than 5×10^−8^. We then repeated steps one and two, such that a single SNP was added to the multiple regression model at each iteration. The algorithm was stopped when no more SNP passed the P<5×10^−8^ requirement.

### Polygene Analysis

To test whether multiple genetic variants of individually weak effect could explain a substantial fraction of sICAM-1 variance, we performed a “polygene” experiment as previously described [42]. Briefly, we randomly divided our dataset in 5 equal parts. We then tested SNPs for association with sICAM-1 using 4 out the 5 parts and performed linkage disequilibrium pruning as implemented in PLINK (r^2^>0.05 and distance <1 Mb). We then derived a gene score with non-redundant associated SNPs using varying P-value thresholds and weighting each SNP for its beta coefficient. Finally, we tested the gene score for association with sICAM-1 in the remaining one fifth of the total sample and calculated the adjusted R^2^. This experiment was repeated 5 times using each one of the five parts as the gene score validation group alternatively.

### Replication of Novel Associations in CHARGE

We sought to replicate the 4 novel loci identified in 9,813 individuals from the Cohorts for Heart and Aging Research in Genome Epidemiology (CHARGE) consortium [14] for whom plasma sICAM-1 concentration and genotypes were available. The CHARGE sample consists of 4 meta-analyzed cohorts: the Framingham Heart Study, the Cardiovascular Health Study, the Atherosclerosis Risk in Communities study, and the Rotterdam Study. Complete information on each study is available as Text S1. Association analyses were performed on imputed genotypes using an additive genetic model on age and sex adjusted log-transformed sICAM-1 values.

## Supporting Information

Table S1 Genome-wide significant associations with sICAM-1.
(0.12 MB DOC)

Table S2 Clinical characteristics of WGHS.
(0.03 MB DOC)

Text S1 Description of study cohorts.
(0.05 MB DOC)

## References

[1] van de Stolpe A, van der Saag PT (1996). Intercellular adhesion molecule-1.. J Mol Med.

[2] Ridker PM, Hennekens CH, Roitman-Johnson B, Stampfer MJ, Allen J (1998). Plasma concentration of soluble intercellular adhesion molecule 1 and risks of future myocardial infarction in apparently healthy men.. Lancet.

[3] Pradhan AD, Rifai N, Ridker PM (2002). Soluble intercellular adhesion molecule-1, soluble vascular adhesion molecule-1, and the development of symptomatic peripheral arterial disease in men.. Circulation.

[4] Song Y, Manson JE, Tinker L, Rifai N, Cook NR (2007). Circulating levels of endothelial adhesion molecules and risk of diabetes in an ethnically diverse cohort of women.. Diabetes.

[5] Pare G, Chasman DI, Kellogg M, Zee RY, Rifai N (2008). Novel association of ABO histo-blood group antigen with soluble ICAM-1: results of a genome-wide association study of 6,578 women.. PLoS Genet.

[6] Barbalic M, Dupuis J, Dehghan A, Bis JC, Hoogeveen RC (2010). Large-scale genomic studies reveal central role of ABO in sP-selectin and sICAM-1 levels.. Hum Mol Genet.

[7] Qi L, Cornelis MC, Kraft P, Jensen M, van Dam RM (2010). Genetic variants in ABO blood group region, plasma soluble E-selectin levels and risk of type 2 diabetes.. Hum Mol Genet.

[8] Bielinski SJ, Pankow JS, Foster CL, Miller MB, Hopkins PN (2007). Circulating soluble ICAM-1 levels shows linkage to ICAM gene cluster region on chromosome 19: The NHLBI Family Heart Study follow-up examination..

[9] Kent JW, Mahaney MC, Comuzzie AG, Goring HH, Almasy L (2007). Quantitative trait locus on Chromosome 19 for circulating levels of intercellular adhesion molecule-1 in Mexican Americans.. Atherosclerosis.

[10] Ponthieux A, Lambert D, Herbeth B, Droesch S, Pfister M (2003). Association between Gly241Arg ICAM-1 gene polymorphism and serum sICAM-1 concentration in the Stanislas cohort.. Eur J Hum Genet.

[11] Puthothu B, Krueger M, Bernhardt M, Heinzmann A (2006). ICAM1 amino-acid variant K469E is associated with paediatric bronchial asthma and elevated sICAM1 levels.. Genes Immun.

[12] Purcell S, Daly MJ, Sham PC (2007). WHAP: haplotype-based association analysis.. Bioinformatics.

[13] Purcell S, Neale B, Todd-Brown K, Thomas L, Ferreira MA (2007). PLINK: a tool set for whole-genome association and population-based linkage analyses.. Am J Hum Genet.

[14] Psaty BM, O'Donnell CJ, Gudnason V, Lunetta KL, Folsom AR (2009). Cohorts for Heart and Aging Research in Genomic Epidemiology (CHARGE) Consortium: Design of prospective meta-analyses of genome-wide association studies from 5 cohorts.. Circ Cardiovasc Genet.

[15] Johansson LE, Lindblad U, Larsson CA, Rastam L, Ridderstrale M (2008). Polymorphisms in the adiponutrin gene are associated with increased insulin secretion and obesity.. Eur J Endocrinol.

[16] Pare G, Cook NR, Ridker PM, Chasman DI (2010). On the use of variance per genotype as a tool to identify quantitative trait interaction effects: a report from the Women's Genome Health Study.. PLoS Genet.

[17] Thorgeirsson TE, Gudbjartsson DF, Surakka I, Vink JM, Amin N (2010). Sequence variants at CHRNB3-CHRNA6 and CYP2A6 affect smoking behavior.. Nat Genet.

[18] Tobacco and Genetics Consortium (2010). Genome-wide meta-analyses identify multiple loci associated with smoking behavior.. Nat Genet.

[19] Liu JZ, Tozzi F, Waterworth DM, Pillai SG, Muglia P (2010). Meta-analysis and imputation refines the association of 15q25 with smoking quantity.. Nat Genet.

[20] Xu Z, Taylor JA (2009). SNPinfo: integrating GWAS and candidate gene information into functional SNP selection for genetic association studies.. Nucleic Acids Res.

[21] Collins T, Read MA, Neish AS, Whitley MZ, Thanos D (1995). Transcriptional regulation of endothelial cell adhesion molecules: NF-kappa B and cytokine-inducible enhancers.. FASEB J.

[22] Ledebur HC, Parks TP (1995). Transcriptional regulation of the intercellular adhesion molecule-1 gene by inflammatory cytokines in human endothelial cells. Essential roles of a variant NF-kappa B site and p65 homodimers.. J Biol Chem.

[23] Romeo S, Kozlitina J, Xing C, Pertsemlidis A, Cox D (2008). Genetic variation in PNPLA3 confers susceptibility to nonalcoholic fatty liver disease.. Nat Genet.

[24] Yuan X, Waterworth D, Perry JR, Lim N, Song K (2008). Population-based genome-wide association studies reveal six loci influencing plasma levels of liver enzymes.. Am J Hum Genet.

[25] Kantartzis K, Peter A, Machicao F, Machann J, Wagner S (2009). Dissociation between fatty liver and insulin resistance in humans carrying a variant of the patatin-like phospholipase 3 gene.. Diabetes.

[26] Velazquez L, Cheng AM, Fleming HE, Furlonger C, Vesely S (2002). Cytokine signaling and hematopoietic homeostasis are disrupted in Lnk-deficient mice.. J Exp Med.

[27] Newton-Cheh C, Johnson T, Gateva V, Tobin MD, Bochud M (2009). Genome-wide association study identifies eight loci associated with blood pressure..

[28] Levy D, Ehret GB, Rice K, Verwoert GC, Launer LJ (2009). Genome-wide association study of blood pressure and hypertension..

[29] Gudbjartsson DF, Bjornsdottir US, Halapi E, Helgadottir A, Sulem P (2009). Sequence variants affecting eosinophil numbers associate with asthma and myocardial infarction.. Nat Genet.

[30] Hunt KA, Zhernakova A, Turner G, Heap GA, Franke L (2008). Newly identified genetic risk variants for celiac disease related to the immune response.. Nat Genet.

[31] Todd JA, Walker NM, Cooper JD, Smyth DJ, Downes K (2007). Robust associations of four new chromosome regions from genome-wide analyses of type 1 diabetes.. Nat Genet.

[32] Talmud PJ, Drenos F, Shah S, Shah T, Palmen J (2009). Gene-centric association signals for lipids and apolipoproteins identified via the HumanCVD BeadChip.. Am J Hum Genet.

[33] Soranzo N, Spector TD, Mangino M, Kuhnel B, Rendon A (2009). A genome-wide meta-analysis identifies 22 loci associated with eight hematological parameters in the HaemGen consortium.. Nat Genet.

[34] Ganesh SK, Zakai NA, van Rooij FJ, Soranzo N, Smith AV (2009). Multiple loci influence erythrocyte phenotypes in the CHARGE Consortium.. Nat Genet.

[35] Ridker PM, Chasman DI, Zee RY, Parker A, Rose L (2008). Rationale, Design, and Methodology of the Women's Genome Health Study: A Genome-Wide Association Study of More Than 25 000 Initially Healthy American Women.. Clin Chem.

[36] Register TC, Burdon KP, Lenchik L, Bowden DW, Hawkins GA (2004). Variability of serum soluble intercellular adhesion molecule-1 measurements attributable to a common polymorphism.. Clin Chem.

[37] Eschen O, Christensen JH, Dethlefsen C, Schmidt EB (2008). Cellular Adhesion Molecules in Healthy Subjects: Short Term Variations and Relations to Flow Mediated Dilation.. Biomark Insights.

[38] Wigginton JE, Cutler DJ, Abecasis GR (2005). A note on exact tests of Hardy-Weinberg equilibrium.. Am J Hum Genet.

[39] Price AL, Patterson NJ, Plenge RM, Weinblatt ME, Shadick NA (2006). Principal components analysis corrects for stratification in genome-wide association studies.. Nat Genet.

[40] Frazer KA, Ballinger DG, Cox DR, Hinds DA, Stuve LL (2007). A second generation human haplotype map of over 3.1 million SNPs.. Nature.

[41] Pe'er I, Yelensky R, Altshuler D, Daly MJ (2008). Estimation of the multiple testing burden for genomewide association studies of nearly all common variants.. Genet Epidemiol.

[42] Purcell SM, Wray NR, Stone JL, Visscher PM, O'Donovan MC (2009). Common polygenic variation contributes to risk of schizophrenia and bipolar disorder.. Nature.

[43] McVean GA, Myers SR, Hunt S, Deloukas P, Bentley DR (2004). The fine-scale structure of recombination rate variation in the human genome.. Science.

[44] Winckler W, Myers SR, Richter DJ, Onofrio RC, McDonald GJ (2005). Comparison of fine-scale recombination rates in humans and chimpanzees.. Science.

