# Supplementary material for: Genome-Wide Association Analysis of Soluble ICAM-1 Concentration Reveals Novel Associations at the NFKBIK, PNPLA3, RELA, and SH2B3 Loci
Source: PLoS Genet. 2011 Apr 21;7(4):e1001374. doi: 10.1371/journal.pgen.1001374 (PMC3080865; doi:10.1371/journal.pgen.1001374)
Supplement: Table S1 — Genome-wide significant associations with sICAM-1. (0.12 MB DOC) [file pgen.1001374.s001.doc]

| **SNP** | **Chr.** | **Position (Kb)** | **Association P** | **Association Beta** | **MAF** | **H-W P** | **Function** | **Nearest Gene** |
| --- | --- | --- | --- | --- | --- | --- | --- | --- |
| rs6597604 | 9q34.2 | 135039.6 | 1.2E-14 | -5.78 | 0.29 | 0.22 | - | GBGT1 |
| rs8176742 | 9q34.2 | 135121.3 | 3.1E-12 | 5.73 | 0.23 | 0.82 | intron | ABO |
| rs8176740 | 9q34.2 | 135121.3 | 1.4E-12 | 5.75 | 0.24 | 0.40 | intron | ABO |
| rs7873522 | 9q34.2 | 135121.7 | 4.8E-22 | 7.00 | 0.34 | 0.39 | intron | ABO |
| rs8176732 | 9q34.2 | 135122.1 | 1.2E-12 | 5.72 | 0.24 | 0.91 | intron | ABO |
| rs8176731 | 9q34.2 | 135122.2 | 4.5E-21 | 6.89 | 0.34 | 0.82 | intron | ABO |
| rs2073824 | 9q34.2 | 135122.5 | 3.7E-21 | 6.84 | 0.34 | 0.75 | intron | ABO |
| rs2073825 | 9q34.2 | 135122.5 | 7.9E-13 | 5.75 | 0.24 | 0.76 | intron | ABO |
| rs8176720 | 9q34.2 | 135122.7 | 1.3E-21 | 6.92 | 0.34 | 0.68 | intron | ABO |
| rs8176715 | 9q34.2 | 135123.0 | 1.2E-11 | 4.68 | 0.42 | 0.00172 | intron | ABO |
| rs512770 | 9q34.2 | 135123.3 | 2.9E-10 | 5.32 | 0.21 | 0.40 | intron | ABO |
| rs4962040 | 9q34.2 | 135123.4 | 5.7E-14 | 5.25 | 0.39 | 0.06 | intron | ABO |
| rs641943 | 9q34.2 | 135123.5 | 3.7E-16 | 6.33 | 0.26 | 0.57 | intron | ABO |
| rs514708 | 9q34.2 | 135123.6 | 6.4E-16 | 6.28 | 0.26 | 0.59 | intron | ABO |
| rs549446 | 9q34.2 | 135125.1 | 1.1E-12 | 5.73 | 0.24 | 0.61 | intron | ABO |
| rs574347 | 9q34.2 | 135125.5 | 6.2E-13 | 5.77 | 0.24 | 0.77 | intron | ABO |
| rs8176702 | 9q34.2 | 135126.0 | 1.1E-13 | 5.26 | 0.40 | 0.79 | intron | ABO |
| rs2073826 | 9q34.2 | 135126.8 | 5.7E-17 | 5.83 | 0.42 | 0.37 | intron | ABO |
| rs687621 | 9q34.2 | 135126.9 | 4.2E-52 | -10.90 | 0.34 | 0.04 | intron | ABO |
| rs687289 | 9q34.2 | 135126.9 | 1.6E-52 | -10.95 | 0.34 | 0.04 | intron | ABO |
| rs2073827 | 9q34.2 | 135127.0 | 2.8E-17 | 5.88 | 0.41 | 0.00269 | intron | ABO |
| rs2073828 | 9q34.2 | 135127.0 | 4.3E-17 | 5.83 | 0.42 | 0.15 | intron | ABO |
| rs8176694 | 9q34.2 | 135127.5 | 3.4E-12 | 6.24 | 0.18 | 0.42 | intron | ABO |
| rs657152 | 9q34.2 | 135129.1 | 3.2E-44 | -9.87 | 0.37 | 0.00083 | intron | ABO |
| rs500498 | 9q34.2 | 135138.5 | 8.0E-33 | 8.25 | 0.45 | 0.63 | intron | ABO |
| rs505922 | 9q34.2 | 135139.0 | 3.9E-52 | -10.92 | 0.34 | 0.04 | intron | ABO |
| rs507666 | 9q34.2 | 135139.2 | 3.0E-91 | -17.34 | 0.20 | 0.00072 | intron | ABO |
| rs630014 | 9q34.2 | 135139.5 | 1.5E-22 | 6.71 | 0.47 | 0.73 | intron | ABO |
| rs568203 | 9q34.2 | 135141.3 | 1.4E-12 | 5.70 | 0.24 | 0.83 | - | ABO |
| rs8176635 | 9q34.2 | 135141.8 | 1.5E-12 | 5.16 | 0.33 | 0.52 | - | ABO |
| rs8176634 | 9q34.2 | 135141.9 | 1.3E-09 | 5.34 | 0.19 | 0.53 | - | ABO |
| rs7025162 | 9q34.2 | 135156.2 | 2.0E-09 | 5.34 | 0.18 | 0.14 | - | ABO |
| rs1049728 | 11q13.1 | 65177.7 | 2.7E-16 | -11.47 | 0.06 | 0.79 | 3' Untranslated | RELA |
| rs3184504 | 12q24.12 | 110369.0 | 2.9E-17 | 5.83 | 0.49 | 0.01 | coding-nonsynonymous | SH2B3 |
| rs653178 | 12q24.12 | 110492.1 | 3.2E-17 | 5.79 | 0.49 | 0.45 | intron | ATXN2 |
| rs11066188 | 12q24.13 | 111095.1 | 4.3E-12 | 4.82 | 0.42 | 0.58 | intron | C12orf51 |
| rs11066320 | 12q24.13 | 111390.8 | 3.9E-10 | 4.34 | 0.43 | 0.76 | intron | PTPN11 |
| rs10404517 | 19p13.2 | 10188.9 | 3.9E-15 | -7.91 | 0.13 | 0.28 | coding-nonsynonymous | N/A |
| rs10409243 | 19p13.2 | 10194.0 | 5.0E-47 | -10.08 | 0.41 | 0.60 | locus | S1PR2 |
| rs2116941 | 19p13.2 | 10195.4 | 8.8E-37 | -10.98 | 0.20 | 0.72 | locus | S1PR2 |
| rs2288937 | 19p13.2 | 10201.9 | 1.1E-21 | 8.65 | 0.17 | 0.73 | intron | S1PR2 |
| rs12462481 | 19p13.2 | 10217.4 | 9.1E-11 | -5.98 | 0.17 | 0.74 | - | MRPL4 |
| rs8111930 | 19p13.2 | 10229.0 | 8.8E-45 | 14.68 | 0.12 | 0.21 | intron | MRPL4 |
| rs1059849 | 19p13.2 | 10231.7 | 1.5E-17 | 5.95 | 0.41 | 0.31 | - | MRPL4 |
| rs5030392 | 19p13.2 | 10249.5 | 1.1E-15 | 26.46 | 0.01 | 0.05 | intron | ICAM1 |
| rs1799969 | 19p13.2 | 10255.8 | 1.3E-120 | -24.91 | 0.11 | 0.50 | coding-nonsynonymous | ICAM1 |
| rs5498 | 19p13.2 | 10256.7 | 5.7E-89 | 13.76 | 0.43 | 0.13 | coding-nonsynonymous | ICAM1 |
| rs923366 | 19p13.2 | 10258.2 | 3.6E-83 | 13.32 | 0.44 | 0.50 | - | ICAM1 |
| rs3093030 | 19p13.2 | 10258.4 | 5.8E-84 | 13.34 | 0.43 | 0.34 | - | ICAM1 |
| rs2569693 | 19p13.2 | 10260.9 | 1.9E-37 | 8.95 | 0.39 | 0.18 | - | ICAM4 |
| rs281440 | 19p13.2 | 10261.3 | 1.6E-52 | -12.61 | 0.22 | 0.77 | - | ICAM5 |
| rs2075741 | 19p13.2 | 10262.1 | 4.6E-83 | 13.28 | 0.44 | 0.43 | intron | ICAM5 |
| rs2228615 | 19p13.2 | 10264.4 | 7.5E-37 | 8.90 | 0.39 | 0.19 | coding-nonsynonymous | ICAM5 |
| rs281423 | 19p13.2 | 10296.5 | 2.4E-18 | -7.86 | 0.17 | 0.17 | intron | RAVER1 |
| rs2230399 | 19p13.2 | 10305.6 | 4.4E-08 | 6.82 | 0.08 | 0.70 | coding-nonsynonymous | ICAM3 |
| rs2278442 | 19p13.2 | 10305.8 | 2.7E-30 | -8.26 | 0.35 | 0.36 | intron | ICAM3 |
| rs2304237 | 19p13.2 | 10307.6 | 4.1E-28 | 9.08 | 0.23 | 0.94 | coding-nonsynonymous | ICAM3 |
| rs281414 | 19p13.2 | 10311.3 | 1.6E-14 | -7.05 | 0.17 | 0.53 | coding-synonymous | ICAM3 |
| rs2304256 | 19p13.2 | 10336.7 | 1.8E-09 | -4.58 | 0.28 | 0.77 | coding-nonsynonymous | TYK2 |
| rs7254474 | 19p13.2 | 10370.4 | 4.2E-16 | 9.44 | 0.10 | 0.05 | intron | CDC37 |
| rs11878850 | 19p13.2 | 10403.6 | 8.7E-13 | 7.49 | 0.12 | 0.09 | - | PDE4A |
| rs1051738 | 19p13.2 | 10438.8 | 1.6E-12 | -6.23 | 0.18 | 0.11 | coding-nonsynonymous | PDE4A |
| rs7256672 | 19p13.2 | 10440.5 | 2.1E-24 | -7.32 | 0.36 | 0.27 | - | PDE4A |
| rs7246953 | 19p13.2 | 10482.1 | 1.6E-08 | -4.96 | 0.19 | 0.04 | - | S1PR5 |
| rs1529729 | 19p13.2 | 11024.6 | 1.1E-10 | 4.44 | 0.46 | 0.73 | intron | SMARCA4 |
| rs738409 | 22q13.31 | 42656.1 | 5.8E-09 | 4.87 | 0.22 | 0.00001 | coding-nonsynonymous | PNPLA3 |
| rs926633 | 22q13.31 | 42668.9 | 8.6E-09 | 5.41 | 0.17 | 0.81 | intron | PNPLA3 |
