# Supplementary material for: Genome-Wide Association Analysis of Soluble ICAM-1 Concentration Reveals Novel Associations at the NFKBIK, PNPLA3, RELA, and SH2B3 Loci
Source: PLoS Genet. 2011 Apr 21;7(4):e1001374. doi: 10.1371/journal.pgen.1001374 (PMC3080865; doi:10.1371/journal.pgen.1001374)
Supplement: Table S2 — Clinical characteristics of WGHS. (0.03 MB DOC) [file pgen.1001374.s002.doc]

| **Characteristic (N=22,435)** | **Mean (SD) or %** |
| --- | --- |
| Age (years) | 54.6 (7.1) |
| BMI (Kg/m^2^) | 25.8 (4.9) |
| sICAM-1 (ng/mL) | 354.8 (80.3) |
| %Smokers | 11.6% |
| %Post-Menopause | 54.0% |
