# Supplementary material for: Genome-Wide Association Analysis of Soluble ICAM-1 Concentration Reveals Novel Associations at the NFKBIK, PNPLA3, RELA, and SH2B3 Loci
Source: PLoS Genet. 2011 Apr 21;7(4):e1001374. doi: 10.1371/journal.pgen.1001374 (PMC3080865; doi:10.1371/journal.pgen.1001374)
Supplement: Text S1 — Description of study cohorts. (0.05 MB DOC) [file pgen.1001374.s003.doc]

**Text S1**

**Description of Study Cohorts**

The **ARIC** (Atherosclerosis Risk in Communities) study is a population-based prospective cohort study of CVD and its risk factors[1]. ARIC includes 15,792 persons aged 45-64 years at baseline (1987-89), randomly chosen from four US communities. Cohort members completed four clinic examinations, conducted approximately three years apart between 1987 and 1998. Soluble ICAM-1 was measured in multiple nested case-cohorts samples[2,3]; stratified sampling was used and the sampling frequencies varied across strata. Weighted analyses were repeated to verify the robustness of results to the sampling design. The results were similar to the unweighted analyses and the results of unweighted analyses are shown throughout. 829 participants of European ancestry with genotypes and sICAM-1 levels were available for this study.

The **CHS** (Cardiovascular Health Study) is a population-based cohort study of risk factors for CHD and stroke in adults ≥65 years conducted across four field centers[4]. The original predominantly European ancestry cohort of 5,201 persons was recruited in 1989-1990 from random samples of the Medicare eligibility lists; subsequently, an additional predominantly African-American cohort of 687 persons were enrolled subsequently for a total sample of 5,888. In CHS, sICAM-1 levels were levels were measured as part of a case-cohort substudy, in which selection was based on the presence or absence of prevalent coronary heart disease at baseline and the incidence of cardiovascular events during CHS follow- up. Weighted analyses were performed to account for the sampling design[5]. In the current project, CHS participated in the analyses of sICAM-1 concentrations with the study sample of 1,487 European ancestry individuals who had both genome-wide genotyping and sICAM-1 available for analysis.

The **FHS** (Framingham Heart Study) started in 1948 with 5,209 participants from Framingham, Massachusetts, US, who have undergone biannual examinations to investigate CVD and its risk factors. In 1971, the Offspring cohort (comprised of 5,124 children of the Original cohort, and the children’s spouses) and in 2002, the Third Generation (consisting of 4,095 children of the Offspring cohort), were recruited. For the current project, the study sample consisted of 6,845 individuals with sICAM-1 concentrations (Offspring and Third Generation). Linear mixed effect models were used to account for family structure of FHS.

The **RS** (Rotterdam Study) is a prospective, community-based cohort study of determinants of several chronic diseases in older adults. In brief, the study comprised 7,983 inhabitants of Ommoord, a district of Rotterdam in the Netherlands, who were 55 years or over[6,7]. The baseline examination took place between 1990 and 1993. For the current project, the study sample consisted of 600 individuals with sICAM-1 concentrations.

**Genotyping and imputation**

**ARIC**: Affymetrix 6.0 array genotypes were obtained in 8,861 self-identified whites: 734 individuals were excluded for the following reasons: 1) discordant with previous genotype data, 2) genotypic sex did not match phenotypic sex, 3) suspected first-degree relative of an included individual based on genome-wide genotype data, 4) genetic outlier (as assessed by average Identity by State (IBS) using PLINK and >8 standard deviations along any of first 10 principal components in EIGENSTRAT after 5 iterations. Imputation of ~2.5 million autosomal SNPs in HapMap with reference to release 22 of the CEU sample was conducted using the algorithm implemented in MACH.

**CHS :** A GWAS was conducted in a subset of CHS participants (n=3,980), all of whom were without clinical CVD at their baseline clinical visit and provided consent to use their DNA for research. Because the participants from the other cohorts were primarily Caucasian or of European-descent, the African-American CHS participants were excluded from these analyses to reduce the possibility of confounding by population stratification. Genotyping was performed using the Illumina 370 CNV BeadChip system. Samples were excluded for sex mismatch, discordance with prior genotyping, or call rate <95%. SNPs were excluded from analysis when monomorphic, when HWE p<10^-5^, and when call rates were <97%. Imputation was performed using BIMBAM v0.99 with reference to HapMap CEU using release 22, build 36.

**FHS:** Genotyping was carried-out as a part of the SHARe project using the Affymetrix 500K mapping array (250K Nsp and 250K Sty arrays) and the Affymetrix 50K supplemental gene focused array on 9,274 individuals. Genotyping resulted in 503,551 SNPs with successful call rate >95% and HWE p>10^-6^ on 8,481 individuals with call rate >97%. Imputation of ~2.5 million autosomal SNPs in HapMap with reference to release 22 CEU sample was conducted using the algorithm implemented in MACH.

**RS**: Genotyping was conducted using the Version 3 Illumina Infinium II HumanHap550. SNPs were excluded for minor allele frequency ≤1% (24,977 SNPs), Hardy-Weinberg equilibrium (HWE) p<10^-5^, or SNP call rate ≤90% (811 SNPs) , resulting in data on 530,683 SNPs. Imputation was done with reference to HapMap release 22 CEU using the maximum likelihood method implemented in MACH.

***ICAM-1 Measurements***

**ARIC:** Circulating ICAM-1 levels were determined by quantitative sandwich ELISA (R&D Systems, Minneapolis, MN ). EDTA plasma samples were run in duplicate and the intra- and inter-assay coefficients of variation were 4.0% and 5.1%, respectively. The minimum detectable dose was 0.35 ng/ml with a standard curve range of 1.6-50 ng/ml. There was a 20-fold sample dilution and the assay range was 32-1000 ng/ml.

**CHS:** sICAM-1 was determined by quantitative ELISA (R&D Systems, Minneapolis, MN). The minimum detectable dose was 0.35 ng/ml with a standard curve range of 1.6-50 ng/ml. There was a 20-fold sample dilution and the assay range was 32-1000 ng/ml. Samples were run in duplicate. The intra-assay coefficient of variation was 5.0±3.5%. The threshold for re-measuring was 1000 ng/ml.

**FHS**: Serum ICAM-1 was measured by quantitative ELISA (R&D Systems (Cat. No. BBE 1B)

[**http://www.rndsystems.com/**](http://www.rndsystems.com/)). The minimum detectable dose is <0.35 ng/ml, standard curve 0 - 50 ng/mL. Samples were run in duplicate. Intra- and inter-assay coefficients of variation were 3.93±2.86 and 3.94±2.97respectively.

**RS:** The levels of sICAM-1 were determined by means of commercially available enzyme-linked immunosorbent assays (sICAM-1 kits; R&D Systems Europe). Interassay coefficients of variation was 6.9%.

**References:**

1. The ARIC investigators (1989) The Atherosclerosis Risk in Communities (ARIC) Study: design and objectives. The ARIC investigators. Am J Epidemiol 129: 687-702.

2. Hwang SJ, Ballantyne CM, Sharrett AR, Smith LC, Davis CE, et al. (1997) Circulating adhesion molecules VCAM-1, ICAM-1, and E-selectin in carotid atherosclerosis and incident coronary heart disease cases: the Atherosclerosis Risk In Communities (ARIC) study. Circulation 96: 4219-4225.

3. Volcik KA, Ballantyne CM, Coresh J, Folsom AR, Wu KK, et al. (2006) P-selectin Thr715Pro polymorphism predicts P-selectin levels but not risk of incident coronary heart disease or ischemic stroke in a cohort of 14595 participants: the Atherosclerosis Risk in Communities Study. Atherosclerosis 186: 74-79.

4. Fried LP, Borhani NO, Enright P, Furberg CD, Gardin JM, et al. (1991) The Cardiovascular Health Study: design and rationale. Ann Epidemiol 1: 263-276.

5. Jenny NS, Arnold AM, Kuller LH, Sharrett AR, Fried LP, et al. (2006) Soluble intracellular adhesion molecule-1 is associated with cardiovascular disease risk and mortality in older adults. J Thromb Haemost 4: 107-113.

6. Hofman A, Breteler MM, van Duijn CM, Krestin GP, Pols HA, et al. (2007) The Rotterdam Study: objectives and design update. Eur J Epidemiol 22: 819-829.

7. Hofman A, Grobbee DE, de Jong PT, van den Ouweland FA (1991) Determinants of disease and disability in the elderly: the Rotterdam Elderly Study. Eur J Epidemiol 7: 403-422.
